# Awareness strategies and the physiological impact of pain in prehospital analgesia management

**DOI:** 10.1186/s13049-025-01343-0

**Published:** 2025-03-05

**Authors:** Neslihan Ergun Suzer, Sarper Yilmaz

**Affiliations:** 1 Kocaeli Darıca Farabi Training and Research Hospital, Kocaeli, Turkey; 2 Dr Lütfi Kırdar Kartal Eğitim ve Araştırma Hastanesi, Istanbul, Turkey

Dear editor,

We read with great interest the study by Larsson et al., titled *“Pain assessment and management of adult patients in the Swedish EMS: a nationwide registry study”* [1]. The study, offering extensive prehospital data from Sweden, serves as an experience-sharing resource for countries seeking to improve pain management in prehospital settings. Such large-scale national studies provide valuable opportunities for various nations to evaluate their systems, identify gaps, and improve key aspects of pain management, including medication administration, the types of analgesics used, delivery methods, and the identification of patients requiring analgesia. Several findings from this study stand out and hold potential for guiding future strategies in prehospital care.

Currently, the Royal College of Emergency Medicine highlights four key issues related to pain management in emergency departments (EDs): (1) the perceived prioritization of the 4-hour waiting time target and ambulance handover over effective pain management; (2) limited individual feedback on pain management and the lack of regular discussions on optimal pain control within EDs; (3) failure to address poor pain management performance, leading staff to believe that pain management is adequately handled; and (4) skepticism among individual staff regarding the usefulness of pain scores [2]. Similar challenges are reflected in the prehospital setting in Sweden, as observed in Larsson et al.’s study. Therefore, there is a need for patient-centered, reliable medications and application methods, supported by objective criteria, to ensure timely pain management and dynamic monitoring in both EDs and prehospital care. This also highlights the need for improved awareness.

In line with this, Yilmaz et al. recently proposed a modification to the START triage system, introducing the START-A (Simple Triage and Rapid Treatment with Analgesia) model for prehospital mass casualty triage [3, 4]. Effective early analgesia requires that prehospital personnel not only be capable of evaluating patients’ clinical presentations but also have sufficient awareness of pain management needs, monitoring requirements, and the necessary knowledge and competency to manage them. A crucial step toward improving this awareness could be the integration of pain assessment into the documentation system used in ambulances. For example, after recording the patient’s demographic information, chief complaint, and vital signs, pain could be assessed using the Visual Analogue Scale (VAS), the Verbal Rating Scale (VRS), or the Numerical Rating Scale (NRS). As highlighted in Larsson et al.’s study, pain scores are closely associated with vital parameters [5].

Another important consideration involves the use of Early Warning Scores (EWS) in prehospital settings to assess patients’ severity and guide clinical decision-making [6]. Given the link between pain and vital parameters, research on whether clinical predictions are more accurate before or after pain management remains limited. Larsson et al.’s finding that heart rate is not significantly affected by pain management is noteworthy. This suggests that EWS systems incorporating heart rate may be less susceptible to pain-related variability, thereby enhancing their predictive accuracy for patient outcomes. This observation underscores the need to re-evaluate the impact of pain on heart rate and other parameters within EWS frameworks.

In summary, developing awareness strategies for prehospital pain management could improve analgesic outcomes, but further research is needed to understand the physiological impact of pain on vital systems. Investigating these dynamics will help refine and optimize prehospital pain.

## Data Availability

No datasets were generated or analysed during the current study.
